# Pathophysiology of Lipid Droplets in Neuroglia

**DOI:** 10.3390/antiox11010022

**Published:** 2021-12-23

**Authors:** Tina Smolič, Robert Zorec, Nina Vardjan

**Affiliations:** 1 Laboratory of Neuroendocrinology-Molecular Cell Physiology, Institute of Pathophysiology, Faculty of Medicine, University of Ljubljana, 1000 Ljubljana, Slovenia; tina.smolic@mf.uni-lj.si (T.S.); robert.zorec@mf.uni-lj.si (R.Z.); 2 Laboratory of Cell Engineering, Celica Biomedical, 1000 Ljubljana, Slovenia

**Keywords:** lipid droplets, neuroglia, astrocytes, metabolic and oxidative stress, neurologic disorders, pathophysiology

## Abstract

In recent years, increasing evidence regarding the functional importance of lipid droplets (LDs), cytoplasmic storage organelles in the central nervous system (CNS), has emerged. Although not abundantly present in the CNS under normal conditions in adulthood, LDs accumulate in the CNS during development and aging, as well as in some neurologic disorders. LDs are actively involved in cellular lipid turnover and stress response. By regulating the storage of excess fatty acids, cholesterol, and ceramides in addition to their subsequent release in response to cell needs and/or environmental stressors, LDs are involved in energy production, in the synthesis of membranes and signaling molecules, and in the protection of cells against lipotoxicity and free radicals. Accumulation of LDs in the CNS appears predominantly in neuroglia (astrocytes, microglia, oligodendrocytes, ependymal cells), which provide trophic, metabolic, and immune support to neuronal networks. Here we review the most recent findings on the characteristics and functions of LDs in neuroglia, focusing on astrocytes, the key homeostasis-providing cells in the CNS. We discuss the molecular mechanisms affecting LD turnover in neuroglia under stress and how this may protect neural cell function. We also highlight the role (and potential contribution) of neuroglial LDs in aging and in neurologic disorders.

## 1. Introduction

The brain is a highly energy-demanding organ. Although representing only 2% of total body mass, it utilizes ~20% of the total O_2_ consumed by the resting body [1]. Lipids represent ~50% of the brain’s dry weight, but energy provision to the brain preferentially relies on a continuous circulatory supply of glucose and oxygen to neural cells; the utilization of energy-rich free fatty acids (FFAs) is less preferred [2]. It is estimated that only ~20% of the total energy consumption of the adult brain originates from the oxidation of FFAs [3,4], predominantly occurring in astrocytes and not neurons [3]. Despite not being extensively used to provide energy to the brain, lipids play a crucial role in the maintenance of normal brain function as structural constituents of neuronal and neuroglial cell membranes and precursors of signaling molecules [5].

In recent years, there has been increasing interest in the study of the biology of lipid droplets (LDs) in the brain [6,7,8,9,10,11]. LDs are dynamic lipid storage organelles in the cell cytoplasm that play a significant role in cellular lipid turnover and stress response [12] by providing substrates for energy metabolism, building blocks for biological membranes, and precursors for signaling molecules in addition to acting as a buffering system against lipotoxicity [13,14]. LDs consist of a central hydrophobic core of neutral lipids, containing triacylglycerols (TAGs), sterol esters (SEs), and acylceramides [15,16], surrounded by a phospholipid monolayer with various peripheral and embedded proteins [17,18,19]. LDs are believed to be formed de novo in the endoplasmic reticulum (ER). Synthesis of neutral lipids (TAGs and SEs) by diacylglycerol acyltransferases (DGAT1 and DGAT2) and acyl-coenzyme A:cholesterol acyltransferases (ACAT1 and ACAT2) leads to the formation of LDs [12,20,21]. During times of need, FFAs are released from TAGs stored in LDs by two pathways: lipolysis or lipophagy [22,23,24,25]. In the process of lipolysis, adipose triglyceride lipase hydrolyzes TAGs to form diacylglycerides (DAGs) and FFAs. DAGs are further hydrolyzed via PKA-responsive hormone-sensitive lipase to monoacylglycerols (MAGs) and FFAs [26]. Finally, MAG lipase converts MAGs to glycerol and FFAs [27]. In the process of lipophagy, a double-membrane autophagosome engulfs LDs or parts of LDs and fuses with lysosomes containing acid hydrolases that degrade LDs to FFAs [25].

Under normal brain physiology, LDs are observed only at low levels [28], but they tend to accumulate in the brain during development, aging, and in pathologic states (i.e., neurodegenerative diseases, cancer, stroke), particularly in neuroglia (astrocytes, microglia) and rarely in neurons [6,7,9,11,29,30]. Although Alois Alzheimer described “adipose saccules” in brain neuroglial cells of patients with dementia in 1907 [31,32], the role of LDs in central nervous system (CNS) pathology has been greatly overlooked until recently. In this review, we address the most recent advances in understanding of the role of LDs in CNS physiology and pathology when cells are exposed to intracellular and extracellular stressors. We highlight the relevance of LDs in neuroglia, particularly in astrocytes. First, we describe recent data on the characteristics (size, sub-cellular localization, mobility) of LDs in astrocytes. We then discuss how LD turnover in astrocytes is affected by the hyperactivity of neurons, metabolic and hypoxic stress, and stress-related adrenergic activation. We also debate the recent advances in LD biology in microglia, oligodendroglia, and ependymal cells. Finally, we highlight the potential contribution of neuroglial and neuronal LDs to aging and neurologic disorders.

## 2. Lipid Droplets in Astrocytes

Astrocytes are an abundant sub-type of neuroglial cells, which are morphologically and functionally heterogeneous cells responsible for the maintenance of CNS homeostasis [33]. They are the key regulators of CNS energy metabolism [34,35]. Astrocytes take up energy substrates from the bloodstream and extracellular space, such as glucose via plasma membrane glucose transporters and FFAs via diffusion and/or via plasma membrane, namely (i) fatty acid (FA) transport proteins (FATP) that belong to the solute carrier 27 protein (SLC27) family (e.g., FATP1, FATP4), (ii) FA translocase (FAT/CD36), and (iii) FA-binding proteins (FABP; e.g., FABP7) (Table 1), and store them in the form of glycogen [36] and LDs [5,37]. In contrast, neurons are not energy-storing cells and do not contain significant pools of glycogen or LDs, so the energy reserves in neurons are rapidly depleted. During intense activity, neurons depend on energy substrates delivered from neuroglial cells, in particular astrocytes and oligodendrocytes [7,8,38,39,40]. The metabolism of glycogen in astrocytes and its relationship with neuronal function has been studied extensively in the past and recently reviewed (see reviews [36,41,42]). In contrast to glycogen metabolism, the importance of LDs as an energy storage organelle in astrocytes has only recently been emphasized and is discussed in the following sections.

### 2.1. Lipid Droplet Size and Sub-Cellular Localization in Astrocytes

It has recently been reported that ~35% of isolated rat cortical astrocytes 24 h after the seeding (~6 × 10^4^ cells/coverslip) contain multiple LDs with an average diameter of ~450 nm (ranging between 0.2 and 1 µm; [37]), comparable with the measurements performed on hepatocytes [22,43], skeletal muscle [44,45], and tumor cell lines [46]. In contrast, adipocytes normally contain a single LD, occupying most of the cytoplasm and ranging up to 100 µm in diameter [47].

**Table 1:** Lipid transporters in astrocytes and neurons and their functions.

Lipid Transporter	Transporter Subtype	Function	
		Astrocyte	Neuron
Fatty acid transport protein (FATP/SLC27)	FATP1/4	Extracellular FFA uptake [7].	Transport of neuronal de novo synthesized FFA to astrocytes [7].
Fatty acid-binding protein (FABP)	FABP7	Binding and internalization of long-chain FFA [48]. Protection from ROS toxicity through induction of LD accumulation [48]. Regulation of dendritic arbor growth, neuronal excitatory synapse formation, and synaptic transmission [49].	/
Fatty acid translocase (FAT/CD36)	FAT	Increased expression upon treatment with amyloid-β [50]. Mediator of stroke-induced astrocyte activation and scar formation [51].	Long-chain FA transport [52,53]. Uptake of saturated and unsaturated long-chain FA in glucosensing neurons of ventromedial hypothalamus [53].
Apolipoprotein (Apo)	ApoE	FA transport from neurons to astrocytes [7,8]. Transport of toxic peroxidized FA from hyperactive neurons to astrocytes [8]. Release of (very-) long-chain saturated FAs from reactive astrocytes [54].	Transport of neuronal de novo synthesized FFA to astrocytes [7]
	ApoD	FA transport from neurons to astrocytes [7,8].	Transport of neuronal de novo synthesized FFA to astrocytes [7].
	ApoJ	Release of (very-)long-chain saturated FAs from reactive astrocytes [54].	n.d.
ATP-binding cassette transporter (ABC transporter)	ABCA1	Export of cholesterol-transporting ApoE particles from astrocytes [55].	Cholesterol efflux via ApoE particles from neurons [55,56]
Low-density lipoprotein receptor (LDL receptor)	LDL receptor	Uptake of cholesterol-transporting ApoE particles [55].	Uptake of cholesterol-transporting ApoE particles [55].
Low-density lipoprotein receptor-related protein 1	LRP1	n.d.	Uptake of cholesterol-containing ApoE particles [55].

Abbreviations: ABC transporter—ATP-binding cassette transporter; Apo—apolipoprotein; FA—fatty acid; FABP—fatty acid-binding protein; FAT—fatty acid translocase; FATP—fatty acid transport protein; FFA—free fatty acid; LD—lipid droplet; LDL—low-density lipoprotein receptor; n.d.—not determined; SLC16—solute carrier transporter 16; SLC27—solute carrier transporter 27; ROS—reactive oxygen species.

In isolated cortical astrocytes, most LDs are clustered in soma near the nucleus in the vicinity of mitochondria and the ER [37], consistent with studies on adipocytes [57,58,59,60] and different cell lines [61,62], whereas in tissue astrocytes, ~50% of all LDs localize in astrocytic processes and ~50% in the soma [37]. The association of LDs with mitochondria most likely enables the direct transport of lipids from LDs to mitochondria for β-oxidation and energy production [61,62,63,64]. Mitochondria may also provide ATP and NADPH to support the synthesis of FAs, TAGs, and glycolytic precursors from the tricarboxylic acid (Krebs) cycle (e.g., citrate) for esterification into TAGs in the ER and for the storage of TAGs in LDs [59,65]. This suggests that mitochondria may contribute not only to LD breakdown but also to LD formation [59,65,66]. LD–ER contact sites in astrocytes are most likely required for de novo LD biogenesis, as suggested for other cell types [67,68,69,70]. The prevention of de novo LD biogenesis in astrocytes by 24-h inhibition of DGAT1 and DGAT2 enzymes reduces the astrocyte cell number by ~40%, suggesting that the turnover of LDs in resting astrocytes is important for the maintenance of the cell proliferative cycle and/or survival [37].

### 2.2. Mobility of Lipid Droplets in Astrocytes

It is now well-accepted that LDs are mobile cellular organelles which can exhibit different types of motility depending on the cell type, cellular state, and/or nutrient availability [71,72]. In resting isolated cortical astrocytes, LDs display relatively limited mobility compared with astroglial secretory vesicles [73]. More than 95% of LDs in resting astrocytes jitter in a confined area with an average velocity of ~60 nm/s and a maximal displacement (MD) of <1 µm within 60 s of analysis, suggesting non-directional motion around a tethered point at the mitochondria or ER. The rest of the LDs (<5%) exhibit directional motion with an average velocity of ~110 nm/s and an MD of >1 µm within 60 s of analysis [37]. Compared to LDs, the average velocity of secretory vesicles (small synaptic-like and peptidergic vesicles) in astrocytes is ~3- to 10-fold higher (~190–650 nm/s [73] versus ~60 nm/s [37], respectively), with the exception of secretory endolysosomes, which exhibit similar average velocity as LDs but ~2-fold higher MD values (~1.0 μm [endolysosomes] versus 0.4 μm [LDs] within 60 s of analysis). As reported in other cell types, the directional mobility observed in 5% of all LDs in astrocytes is most likely achieved by the motion of LDs via molecular motors along cytoskeletal filaments, i.e., myosins and kinesins/dyneins along actin filaments and microtubules, respectively [71,74]. The directional mobility of LDs might be required for the transport of LDs to (1) different organelles, including mitochondria, ER, LDs, and peroxisomes, where LDs may attach to organelles via docking proteins [12,75] and exchange lipids and proteins [71], or (2) the plasma membrane, where under certain conditions, LDs can be released from astrocytes, such as upon ATP stimulation [76].

The mobility of LDs in astrocytes is reduced by an acute increase in intracellular Ca^2+^ levels upon ATP stimulation and prolonged metabolic stress (24-h nutrient deprivation) [37]. This may strengthen LD–organelle attachment, enhancing the transport of lipids or proteins from the LDs to the attached organelles and vice versa. In Vero cells, it has been shown that acute nutrient deprivation, which activates the energy sensor AMPK, triggers the relocation of LDs and mitochondria along microtubules, enhancing LD–mitochondria interaction and the transfer of FFAs from LDs to mitochondria for energy production [61]. Whether acute nutrient deprivation increases the mobility and delivery of LDs to cell organelles in astrocytes needs to be investigated in the future.

### 2.3. Astrocyte–Neuron Metabolic Coupling and Lipid Droplet Metabolism in Astrocytes

In the CNS, astrocytes and neurons operate as a tightly coupled metabolic unit [77]. During intense activity, neurons rely on astroglial-derived energy substrates, such as L-lactate, produced in astrocytes from blood-derived and/or glycogen-stored glucose in the process of aerobic glycolysis [35,78,79]. Astroglial L-lactate shuttles to neurons through monocarboxylate transporters (MCTs, which belong to the SLC16 family) and lactate channels in the plasma membrane of neurons and astrocytes [80,81]. Lactate is converted to pyruvate in neurons and used as a fuel in oxidative metabolism in mitochondria [82]. In a recent study using primary neuronal–glial co-cultures and *Drosophila* photoreceptor neurons and neuroglia, it has been suggested that, in neurons, glial L-lactate can also serve as a substrate for de novo biogenesis of FFAs, particularly in cells with mitochondrial dysfunction and increased levels of reactive oxygen species (ROS), although astrocyte-neuron lactate shuttle (ANLS)-mediated de novo lipogenesis may also occur under physiologic conditions with functional mitochondria [6,7,8]. FFAs synthesized de novo are then transferred from neurons to astrocytes by FATP1 and 4 and apolipoprotein (Apo) E and ApoD (Table 1) and incorporated into LDs in neuroglia [7]. The transport of FFAs from neurons to astrocytes via ApoE particles was also later confirmed in an astroglial–neuronal co-culture system and in vivo in an acute stroke model, where acute stroke and oxidative stress were induced with a pial strip lesion in the rat brain cortex. The FFA shuttle from neurons to astrocytes in this study was induced by hyperactive neurons and glutamate excitotoxity. The study suggests that astroglial uptake of FFA-loaded ApoE particles most likely occurs via endocytosis [8]. Consistent with these data, primary and rat brain tissue astrocytes, when exposed to excess extracellular FFA, such as oleic acid, are able to take up and accumulate FFAs in LDs [37,83]. According to the study of Liu et al. [7], the inability of ApoE to transport FFA from neurons to glial cells and the inhibition of ANLS during increased ROS levels decrease neuroglial LD accumulation, which leads to neurodegeneration.

Neurons have a low capacity to form LDs and to use FFAs in mitochondrial β-oxidation for ATP production. Compared with glucose metabolism, β-oxidation of FFAs generates more superoxide, the precursor to most other ROS, which, in neurons, with a poor antioxidative defense system, can lead to the accumulation of ROS and oxidative stress [2]. Despite a low capacity to metabolize FFAs, neurons accumulate ROS during intense activity because of active oxidative glucose/lactate metabolism [84]. This can lead to ROS-mediated peroxidation of membrane FAs (as well as protein carbonylation and DNA base oxidation) [85,86,87,88]. Unless neurons remove peroxidized FAs by transporting them to glial cells, which can form LDs from neuron-derived FFAs and possess a strong antioxidative response system [8,89], this can lead to neuronal cell death by apoptosis and neurodegeneration [90]. Astrocytes seem to protect the brain by taking up excess FAs released from neurons and storing them in LDs.

Recently, it has been shown that in response to neuronal activity (e.g., glutamate release), astrocytes break down LDs and consume FFAs by mitochondrial oxidation, which has been suggested to occur in the brain predominantly in astrocytes [3,8,91]. Mobilization of FFAs from LDs for mitochondrial β-oxidation can be achieved by cytosolic lipases [25,28,92] or by autophagosomal degradation of LDs [22,25,93]. However, which of these pathways prevails in astrocytes needs to be determined in the future. Neuronal activity also upregulates the expression of genes in astrocytes involved in energy metabolism and the neutralization of ROS to prevent lipid peroxidation and toxicity [8].

Not only neurons but also astrocytes can release lipids that can be taken up by neurons via endocytosis of ApoE particles or FA transporters (Table 1). Astrocytes can synthesize lipids (even de novo) (e.g., FAs, cholesterol) and ketone bodies and can support neurons. De novo synthesized FAs (e.g., oleic acid, docosahexaenoic acid, arachidonic acid) or FAs released from astrocytic membranes by phospholipase A_2_ can enter neurons via FA transporters or via Apo particles (Table 1) [94,95,96]. Astroglial-derived FAs are mostly used in neurons as building blocks of membranes, which promotes axonal growth and drives neuronal exocytosis, as recently reviewed [5,94,97]. Cholesterol, in the form of cholesterol-carrying lipoproteins unable to pass the blood–brain barrier, is primarily produced in the brain by astrocytes, then serving in synaptogenesis supporting neurons [98]. Cholesterol is exported from astrocytes to neurons via ApoE particles. The latter are endocytosed by neurons via receptors of the low-density lipoprotein (LDL) receptor family (Table 1) and processed in the endosomal/lysosomal system to release cholesterol, which is used in neurons as a membrane component, influencing membrane fluidity, curvature, ion channel function, and synaptogenesis [98,99]. In times of glucose deprivation, ketone bodies, synthesized in astrocytes, can be shuttled from astrocytes to neurons via MCTs, where they are metabolized in mitochondria for energy production [100].

In contrast to the beneficial role of lipids and ketone bodies released from astrocytes, it has been recently revealed that (very-) long-chain saturated FFAs released from reactive astrocytes in ApoE and ApoJ particles can mediate oligodendrocyte and neuronal toxicity, possibly by inducing lipoapoptosis. The authors suggest that this is a novel mechanism by which reactive astrocytes can kill cells in the CNS in neurodegenerative diseases [54].

**Figure 1:**
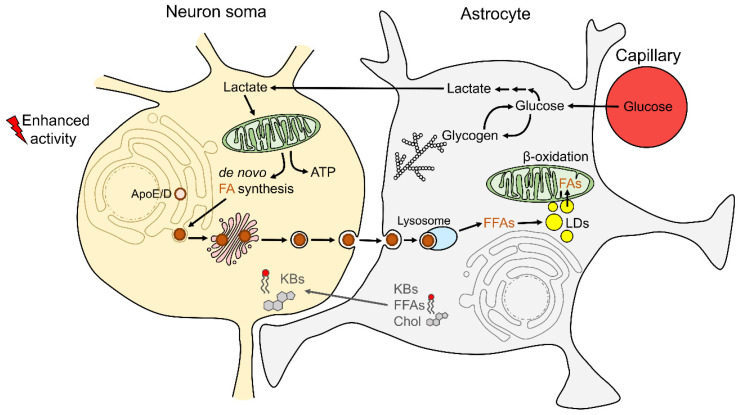
Astrocyte–neuron coupling of lipid metabolism. During enhanced neuronal activity, L-lactate (lactate), produced in astrocytes from glucose in the process of aerobic glycolysis, is transported to neurons via monocarboxylate transporters (MCTs). In neurons, lactate is used in oxidative metabolism for ATP production and/or for de novo fatty acid (FA) synthesis. To avoid the toxicity of free FAs (FFAs), neurons release FFAs in apolipoprotein A/D (ApoE/D) particles, which can enter astrocytes via endocytosis. Once in astrocytes, FFAs are released from ApoE/D particles and are incorporated into lipid droplets (LDs). FAs released from LDs can be further used in astrocytes as a fuel in mitochondrial β-oxidation. Furthermore, in times of starvation, fasting, or intense exercise, astrocytes metabolize FFAs in the process of β-oxidation to produce ketone bodies (KBs) and shuttle them via MCTs to neuronal mitochondria for ATP synthesis, supporting neuronal energy metabolism. Astrocytes also synthesize lipids (FFAs and cholesterol (Chol)) de novo and/or release membrane-bound FAs and transfer them to neurons via Apo particles or FA transporters to be used as membrane components, supporting synaptic membranes and signaling.

Taken together, recent studies strongly suggest that during intense neuronal activity, neurons and astrocytes are metabolically coupled with respect to glucose and lipid metabolism (Figure 1). Neurons take up astroglial lactate for ATP production, which at the same time drives de novo biogenesis of FFAs in neurons and FA transport to astrocytes, where FAs are packed in LDs (to prevent FFA toxicity) and used in mitochondrial β-oxidation for ATP production. Such metabolic interplay between astrocytes and neurons may exist with the mission to provide sufficient energy for both cell types during times of intense CNS activity and, at the same time, prevent oxidative stress that accompanies increased oxidative metabolism in neurons.

### 2.4. Lipid Droplets in Astrocytes and Nutrient Deprivation

**Figure 2:**
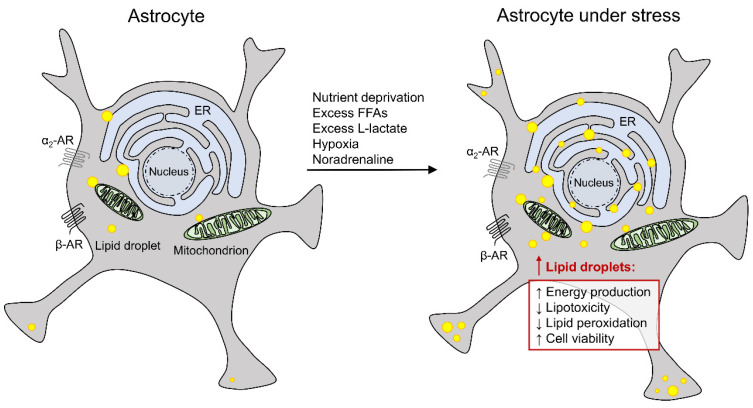
Astrocytes under stress accumulate lipid droplets. Most lipid droplets in astrocytes are in contact with or in close proximity to mitochondria and the endoplasmic reticulum (ER). Metabolic stress (nutrient deprivation, excess free fatty acids [FFAs] or L-lactate), hypoxic stress, and noradrenaline, which activates the CNS stress response via activation of α_2_- and β-adrenergic receptors (ARs), increase the accumulation of lipid droplets in astrocytes. Accumulation of lipids in LDs in stressed cells promotes astrocyte energy production via mitochondrial β-oxidation and prevents FFA-induced lipotoxicity and ROS-mediated membrane lipid peroxidation, which increases the viability of cells under stress (adapted from Smolič et al. [37]).

Recent studies have shown that astrocytes in brain tissue and isolated astrocytes that are cut off from neural cell signals respond to metabolic stress induced by prolonged starvation (24 h) with LD accumulation (Figure 2), increasing the number and the size of LDs [37,101], consistent with starvation-induced LD accumulation observed in other cell types [102,103]. The inhibition of DGAT1 and DGAT2 enzymes, responsible for the final steps of TAG synthesis during LD biogenesis, prevents starvation-induced LD accumulation in primary astrocytes, indicating that starvation induces de novo biogenesis of LDs in astrocytes [37]. Astrocytes under starvation most likely transform their membrane structural components into FFAs, a process dependent on group VIA phospholipase A_2_ [104], which is initially stored in LDs, to prevent cytotoxic effects of the released FFAs, but at the same time, LDs act as a nutrient reservoir. FFAs are believed to be released from LDs at LD–mitochondria contact sites and used for energy production in β-oxidation [62,101,105]. During glucose deprivation, astrocytes most likely switch from glucose to lipid metabolism to increase their viability, as has been shown in a glioma LN18 cell line [101], also increasing neuronal viability because any remaining glucose can be used in neurons as an energy source. In contrast to astrocytes, neurons cannot use FFAs efficiently as a fuel because they cannot contain the mitochondrial overproduction of ROS during β-oxidation [2,4,91]. Under prolonged glucose starvation, FFAs stored in astroglial LDs can also be converted into ketone bodies, which can be transported to neurons as an alternative energy source [106,107], thus increasing neuronal viability in times of prolonged glucose deprivation.

### 2.5. Lipid Droplets in Astrocytes and Excess Extracellular Free Fatty Acids

Astrocytes respond to excess extracellular FFAs (e.g., oleic acid; Figure 2) with the accumulation of LDs [37,83], consistent with observations in some other non-adipocyte cell types [108,109,110,111,112]. The presence of DGAT inhibitors suppresses oleic acid-induced LD accumulation in primary cortical astrocytes, suggesting that the latter is a consequence of de novo biogenesis of LDs from extracellular space-derived FFAs [83]. Extracellular FFAs are most likely taken up by astrocytes via FATP and/or diffusion and stored in LDs to protect astrocytes from lipotoxic stress. Excess FFAs can induce oxidative and ER stress in non-adipocyte cells through alterations in organelle membrane structure and function, production of toxic metabolites, and activation of signaling pathways, leading to cell death, whereas the packing of excess FFAs in LDs has been shown to prevent lipotoxic cell death and be cytoprotective [113].

Given that medium- and long-chain FFAs (i.e., oleic acid, docosahexaenoic acid) are also endogenous ligands of FFA receptor 1 (FFAR1 or GPR40) [114,115] and FFAR4 [116,117] expressed in the brain [114,118,119,120,121], LD accumulation in astrocytes driven by exposure of cells to extracellular FFAs could be regulated via extracellular FFA activation of FFARs and downstream intracellular signaling pathways, as suggested by experiments obtained in Huh-7 cells. Stimulation of Huh-7 cells with oleic acid was shown to activate FFAR4 through a pertussis toxin-sensitive G protein signaling pathway involving phosphoinositide 3-kinase, protein kinase B, and phospholipase D activities, which upregulate LD formation [108]. When inside cells, FFAs can bind to peroxisome proliferator-activated receptors (PPARs) in the nucleus [122]. The expression of PPARs has been confirmed in neural cells, including astrocytes, and linked to the regulation of lipid metabolism [123,124,125]. In rat Sertoli cells, PPARs were shown to regulate the expression of genes that encode proteins involved in FFA transport and metabolism and in LD formation and stabilization [126]; however, whether LD formation in astrocytes is also controlled by the binding of FFAs to PPARs needs to be confirmed in the future.

### 2.6. Lipid Droplets in Astrocytes and Hypoxic Stress

In most CNS pathologies, including ischemia, injury, cancer, and neurodegeneration, besides glucose deprivation, hypoxia also occurs [66,103,127,128,129], favoring anaerobic metabolism with glycolytic production of L-lactate. L-Lactate exits neural cells and may accumulate in extracellular space [130]. In the brain, extracellular L-lactate concentration may vary from ~0.1–1.4 mM to ≥10 mM [131]. Recent studies have shown that prolonged (24 h) exposure of primary and brain tissue astrocytes to increased levels of L-lactate (20 mM) or hypoxia (1% pO_2_) triggers LD accumulation in astrocytes [37] (Figure 2).

The mechanisms underlying LD accumulation in astrocytes on exposure to excess L-lactate need to be determined in the future. One possibility is that increased levels of L-lactate in the brain trigger the accumulation of LDs via activation of L-lactate receptors that have been recently identified on the surface of brain cells, including astrocytes and neurons [132,133,134]. For instance, activation of G_i_ protein-coupled L-lactate-sensitive GPR81 receptor, which downregulates cAMP production and inhibits the activity of cAMP-dependent lipolytic enzymes and lipolysis, may promote energy storage in LDs, as has been shown in myotubes [135] and adipocytes [136,137]. L-Lactate can also enter astrocytes through MCTs and channels [80,138], where it could act as a substrate for de novo FFA synthesis, as shown in neurons [7,8] and oligodendrocytes [139]. This could trigger LD accumulation in astrocytes to protect them from overload of FFAs [6,7,8,29].

Chronic hypoxia may increase the levels of ROS, which hinders enzymatic antioxidant defense systems (e.g., glutathione peroxidase, superoxide dismutase, catalase), leading to oxidative stress and ultimately cell death [15,140]. In neuroglial cells, oxidative stress can induce the accumulation of LDs which act as an antioxidant defense. By actively relocating membrane phospholipids, especially polyunsaturated FAs (PUFAs), which are especially receptive to ROS-induced oxidation, as TAGs into LDs, LDs protect PUFAs from the harmful effects of peroxidation, as shown in *Drosophila* neural stem cells [29].

Although the molecular mechanisms underlying the ROS-mediated redistribution of membrane FFAs to LDs are not yet clear in astrocytes, various studies on other cells implicate the involvement of hypoxia inducible factor 1 (HIF-1) and HIF-2 pathways in astrocytes [141], which can activate the expression of the proteins involved in lipid transport and metabolism, as observed in tumors, myocytes, and other cell types [142,143,144,145]. This may lead to an increase in lipid uptake and trafficking, FFA and sterol synthesis, TAG synthesis, LD biogenesis, and lipid signaling, but an inhibition of lipolysis and β-oxidation [146]. Consistent with this, HIF-inducible fatty acid-binding protein 7 (FABP7) [146], which belongs to the family of FABPs, cellular FFA chaperones controlling FFA uptake and distribution [147], was recently proposed to be involved in the protection of astrocytes from ROS toxicity under hypoxic conditions by mediating an increase in LD accumulation [48]. Moreover, hypoxia may also trigger anaerobic glycolysis in astrocytes and excess production of L-lactate, which can also contribute to LD accumulation, as discussed above.

Chronic exposure of adult and larvae *Drosophila* to a combination of hypoxia and starvation triggers the accumulation of LDs in the *Drosophila* brain, indicating a systemic response to hypoxia and starvation that affects lipid metabolism in the brain. LDs were shown to associate with neuroglial cells, suggesting the involvement of glial cells in hypoxia- and starvation-induced LD accumulation in the *Drosophila* brain [29,37]. Hypoxia and starvation could trigger (1) the translocation of FFAs to brain LDs from the *Drosophila* fat body (the lipid storage site that is a functional equivalent to adipose tissue and liver in mammals [148]) or (2) the translocation of FFAs to LDs from neural cell membrane phospholipids. The translocation of FFAs to brain LDs in times of prolonged systemic oxidative/metabolic stress enables the protection of brain FFAs from peroxidation and their storage in LDs as an alternative source of energy [29,37,48,149], thus increasing neural cell viability [102,149].

### 2.7. Lipid Droplets in Astrocytes and Adrenergic Activation

Noradrenaline is a stress-related neuromodulator released from *locus coeruleus* neurons that activates G protein-coupled adrenergic receptors (ARs) on the surface of neural cells and regulates many CNS processes, including brain glucose metabolism [150,151]. Recently, it has been revealed that astrocytes respond to prolonged exposure to noradrenaline with enhanced LD accumulation [37] (Figure 2), suggesting that noradrenaline also regulates CNS lipid metabolism. Although noradrenaline binds to all types of ARs (α_1_-, α_2_-, and β-ARs) [150], only the activation of β- and α_2_-ARs lead to the enhancement of LD accumulation in astrocytes [37]. Consistent with this, α_2_-AR activation with downregulation of cAMP signals in adipocytes inhibits lipolysis and promotes LD accumulation [92,152]. However, in contrast to astrocytes, where prolonged β-AR activation most likely induces LD formation via upregulation of cAMP signals, activation of β-ARs in other cell types (e.g., adipocytes [92], hepatocytes [153]) enhances lipolysis and LD degradation by activating cAMP-dependent protein kinase A. Protein kinase A then activates lipolysis by phosphorylation of adipose triglyceride lipase, hormone-sensitive lipase, and perilipin-1. These discrepancies between astrocytes and other cell types could be explained by the glycolytic metabolic profile of astrocytes. Despite normal oxygen levels, the activation of β-ARs in astrocytes upregulates glycogenolysis and aerobic glycolysis with L-lactate production [151,154]. As previously discussed, L-lactate could trigger de novo FFA synthesis and therefore LD accumulation in astrocytes, as has been described in neurons [7] and oligodendrocytes [139]. Moreover, cAMP response element-binding protein (CREB), a transcriptional factor that upregulates some genes involved in lipid metabolism in astrocytes, but not in neurons, is activated by β-AR/cAMP signaling in astrocytes [155], which can selectively increase LD accumulation only in astrocytes.

## 3. Lipid Droplets in Microglia

Microglia are heterogeneous macrophage-like cells in the CNS with many homeostatic functions [156]. They are involved in the phagocytic removal of debris and pathogens, antigen presentation, and the innate inflammatory response involving cytokine release and T cell activation, but they can also contribute to synaptic organization and trophic neuronal support during development [157,158]. Microglia are motile cells, constantly extending and retracting their processes to sense environmental changes and interact with neurons and non-neuronal cells. Under pathologic conditions and/or during aging, microglia transform from resting cells, which are highly ramified, to reactive cells with an amoeboid appearance [158]. When activated, they change their transcriptional profile and gain novel functions [159] and may accumulate LDs [9,30]. Depending on the CNS state (e.g., inflammation, neurodegeneration, aging), LD-accumulating reactive microglia were shown to either protect the CNS against disease [30] or contribute to the CNS disease [9].

Lipopolysaccharide (LPS), a bacterial endotoxin which is an innate TLR4 ligand and a proinflammatory microglial stimulus that activates microglia, triggers LD accumulation in microglia in vitro and in situ in tissue cultures by increasing the LD number and size [9,30]. LPS-mediated LD accumulation in microglia most likely involves the activation of the p38 α/β and PI3K/Akt pathways, increasing the expression of perilipin-2, an LD-associated protein, and the colocalization of LDs with cytosolic PLA_2_, a key enzyme for arachidonic acid release [30]. Thus, LDs in LPS-activated microglia likely serve as platforms for the synthesis of inflammatory signaling molecules, the eicosanoids, such as arachidonic acid, contributing to an inflammatory response and neuroprotection in the brain [30]. The increased presence of LDs and perilipin-2 in the CNS may be considered as a biomarker of CNS inflammation in the future [30], as has been suggested for cancer cells [160,161].

Recently, another LD-forming population of reactive microglia has been identified in aged animals and in some neurodegenerative disease mouse models, termed “lipid-droplet-accumulating microglia” (LDAM) [9]. LDAM, compared to neuroprotective LPS-activated LD-rich microglia, have a distinct, only partially overlapping transcriptional profile, which has been linked to microglial dysfunction, such as defects in phagocytosis, excessive secretion of proinflammatory cytokines, production of high ROS levels, and reduction in cholesterol efflux [9]. Thus, dysfunctional and proinflammatory LDAM may contribute to aging and the pathology of neuroinflammation and neurodegeneration. Recently, it has been shown that lipoprotein lipase-deficient microglia upregulate LD accumulation [162]. Reduced lipoprotein lipase levels have been observed in the CNS of patients with Alzheimer’s disease (AD) [163]. Whether reduced lipoprotein lipase levels are linked to the development of dysfunctional LDAM in patients with AD needs to be confirmed in the future.

## 4. Lipid Droplets in Oligodendroglia

Oligodendrocytes are myelin-forming neuroglial cells in the CNS which spirally wrap myelin, a lipid-rich multilayered sheath of membrane, around the axons. This insulates the axons and enables fast saltatory neuronal conduction [164]. LDs were shown to accumulate in immature oligodendrocytes but not in mature oligodendrocytes in vitro, suggesting that during development, when myelin sheaths are formed, lipids that are synthesized de novo in oligodendrocytes are temporarily stored in LDs before they are incorporated into myelin sheaths [165]. LDs were also observed in oligodendrocytes in the brains of aged female mice [166]. During aging or neurodegenerative diseases, myelin sheaths are degraded and myelin-derived FAs can be temporarily stored in LDs before exiting oligodendrocytes and entering astrocytes, where they are transformed into ketone bodies through the process of β-oxidation, to be used as an alternative fuel in neurons [166]. Consistent with this, various studies suggest that during aging and neurodegenerative disease, when glucose hypometabolism is present, the brain switches from glucose to an alternative fuel substrate, ketone bodies [100,167].

## 5. Lipid Droplets in Ependymal Cells

Ependymal cells (e.g., tanycytes) are ciliated epithelial glial cells lined along the surface of the ventricles of the brain and the spinal canal. They build a barrier between the brain parenchyma and cerebrospinal fluid and maintain cerebrospinal fluid homeostasis. Ependymal cells extend their motile cilia into the brain ventricles. Ciliary beating helps to transport nutrients and signaling molecules in the brain ventricles and clear waste products from the brain [168,169,170]. Cilia motility has been linked to the regulation of cerebrospinal fluid flow, brain metabolism [168,169,170], support of neural stem cell proliferation [171], and directional migration of young neurons [172]. Although not typical of other glial cell types, ependymal cells contain a significant number of LDs in normal physiologic conditions. The amount of LDs in ependymal cells, however, increases with age [173,174] and also in animals fed with a high-fat diet [175,176]. Consistent with this, the accumulation of LDs in ependymal cells has been linked to high expression levels of lipid scavenger receptors CD36 and LRP (i.e., LDL receptor-related protein) 1 and 2 [177,178,179]. The role of LDs in ependymal cells with respect to brain function still needs to be determined in the future.

## 6. Lipid Droplets in Neuroglia in Aging and Neurologic Disorders

Although healthy brain tissue usually does not contain excessive amounts of LDs [28], these tend to accumulate in the aged brain, particularly in microglia [9], but can also be observed in astrocytes, ependymal cells, oligodendrocytes, and within neurons [10]. Altered LD metabolism has also been observed in neuroglia in various neurologic disorders and CNS disease models, including neurodegenerative diseases, stroke, and glioma [5,180,181,182]. The molecular mechanisms causing dysregulation of LD metabolism in aging and neurologic disorders are largely unknown, but various recent papers have linked altered LD metabolism in neural cells directly to disease-specific gene mutations and environmental changes, which are discussed next.

Recently, cytoplasmic inclusions of the truncated TAR DNA-binding protein 43 (TDP-43), a pathologic hallmark of the motor neuron neurodegenerative disorder amyotrophic lateral sclerosis (ALS) as well as frontotemporal dementia [183,184,185,186], were shown to trigger the accumulation of LDs in primary cortical astrocytes, suggesting altered lipid metabolism in astrocytes with TDP-43 inclusions, which can affect neuronal function in ALS and frontotemporal dementia given that astrocytes and neurons are a tightly coupled metabolic unit [187].

Moreover, ATL1, SPAST, and REEP1 genes (encoding atlastin-1, spastin, and receptor expression-enhancing protein 1, respectively), the mutations of which cause the neurodegenerative disorder autosomal dominant hereditary spastic paraplegia (HSP) in most cases, were linked to LD metabolism in neurons and/or neuroglial cells [188,189]. The study by Mou et al. [188], conducted on astrocytes and neurons derived from human pluripotent stem cells (hPSCs) with introduced ATL1 missense mutations associated with spastic paraplegia 3A (SPG3A), the most prevalent early-onset autosomal dominant HSP, revealed that SPG3A astrocytes exhibit reduced LD size and disrupt cholesterol transfer to cortical projection neurons, leading to cholesterol deficiency in neurons. Treating SPG3A cortical projection neurons with cholesterol or conditioned medium from control astrocytes rescued axonal degeneration. Thus, impaired lipid metabolism in astrocytes may cause the degeneration of cortical projection neurons, suggesting a non-cell autonomous mechanism leading to neuronal pathology in HSP [188]. It was also shown that downregulation of SPAST decreases the size of LDs in neurons, suggesting that spastin is a positive regulator of LD metabolism in neurons [190]. Moreover, neurons from *Reep1* null mice show fewer and smaller LDs [189]. REEP1 is normally not present on LDs, but the expression of HSP-associated mutant variants of REEP1 in mammalian cells mistargets REEP1 to LDs [191]. Furthermore, mice lacking a major brain triglyceride hydrolase, DDHD2, the mutations of which cause complex HSP, have higher triglyceride levels in the brain and increased LD accumulation in neurons, resulting in cognitive and motor abnormalities typical of complex HSP [192,193]. The observed dysfunction of LDs in neurons caused by HSP-linked gene mutations could also affect glial lipid metabolism, contributing to the pathogenesis of HSP, which needs to be investigated in the future.

Parkinson’s disease (PD) is a neurodegenerative disorder associated with the loss of *substantia nigra* dopaminergic neurons and the intraneuronal accumulation/aggregation of protein α-synuclein in the form of Lewy bodies and Lewy neurites [194,195]. Wild-type α-synuclein has been shown to accumulate on the surface of LDs in primary neurons, suggesting that LDs serve as a platform for α-synuclein deposition. The accumulation of α-synuclein on the surface of LDs protects triglycerides stored in LDs from degradation, facilitating LD accumulation [196,197]. The relevance of the biological interaction between α-synuclein and LDs in PD is not clear, and further research is needed to see if LDs are involved in α-synuclein aggregation and synucleinopathies. Consistent with an implication of LDs in the pathogenesis of PD, reduced content of neutral lipids has been observed in astrocytes in the *substantia nigra* of the brain of patients with PD, whereas neurons and microglia, in contrast to astrocytes, exhibit increased neutral lipid content, suggesting dysregulated lipid homeostasis in PD [198]. Whether PD pathogenesis could be remedied by restoring lipid homeostasis between neurons, astrocytes, and microglia needs to be determined in the future [198].

LDs in neuroglial cells in the postmortem brains of patients with AD, a progressive neurodegenerative disorder that affects memory, cognition, and behavior, were first described by Alois Alzheimer [31,32]. LDs were shown to accumulate in ependymal cells of the 3×Tg-AD mouse model and postmortem AD brains, which are the main support cells of the forebrain neural stem cell niche and may inhibit neural stem cell proliferation and regeneration function [199]. Overexpression of ApoE4, the strongest genetic risk factor for the late onset of AD among the three major ApoE allelic variants (ε2, ε3, ε4) in humans [200,201], has been shown to alter lipid metabolism in astrocytes, increasing the utilization of endogenous FAs and LD formation [180]. ApoE was shown to mediate the transport of toxic peroxidized FAs from hyperactive neuron to astrocytic LDs. Astrocytes then consume FAs stored in LDs by β-oxidation and turn on a detoxification gene expression program [7,8]. Overexpression of the ApoE4 isoform in neurons and astrocytes can reduce the sequestration of neuronal FAs into neuronal LDs, reduce the transfer efficiency of lipids between neurons and astrocytes, and reduce FA β-oxidation. This leads to lipid accumulation and compromises astrocytic metabolic support to neurons and thus neuronal function [202]. However, whether ApoE4-mediated lipid dysregulation in neurons and astrocytes also exists in the brains of patients with AD needs to be determined. Furthermore, using cell sorting with RNA sequencing and lipidomics, it has recently been suggested that a loss of function in the gene encoding for TREM2 (triggering receptor expressed on myeloid cells 2), which is selectively expressed in microglia and like ApoE, has been linked to late onset of AD and causes neuronal damage and the accumulation of cholesterol esters in the *Trem2^−/−^* mouse brain and in isolated *Trem2^−/−^* microglia. *Trem2^−/−^* microglia are capable of phagocytosing cholesterol-rich myelin debris in disease situations but exhibit impairment in cholesterol efflux, which results in the accumulation of myelin-derived cholesteryl esters in microglial LDs, which may contribute to disease progression [203].

LDs were also observed in the striatal tissue of patients with Huntington’s disease (HD) and in primary striatal neurons from a mouse model of HD [204], an inherited neurodegenerative disorder caused by a defective *HTT* gene encoding huntingtin that affects movement, behavior, and cognition [205]. The role of LDs in the pathogenesis of HD and whether LDs are also present in striatal neurons and/or glial cells in the brains of patients with HD still needs to be determined.

LDs can also be detected in infarcted regions of rodent stroke models in microglia [206] and astrocytes [8]. The role of LDs and the mechanisms underlying LD accumulation in stroke are not yet clear, but they may be related to stroke-induced microenvironmental changes, e.g., hypoxia, nutrient deprivation, and excitotoxicity, leading to cellular stress and LD formation to prevent further CNS damage.

LDs are also observed in gliomas [207], the most common type of CNS tumor, derived from neuroglia, i.e., astrocytes, oligodendrocytes, or ependymal cells [103,207,208]. In gliomas, to meet the cell’s energy demands, tumor tissue enhances de novo lipogenesis through the upregulation of SREBP1 and FASN and/or takes up FAs from the circulation for mitochondrial β-oxidation [209,210,211,212,213]. Furthermore, glioma cells exhibit increased cholesterol uptake through LDL receptors that are upregulated by SREBP1 [214]. Cytosolic increase in FFAs and cholesterol can cause ER stress and lipotoxicity, which can lead to cell death [215,216,217]. Therefore, glioma cells store surplus FFAs and cholesterol inside LDs, promoting cell viability and survival [145,207] and therefore tumor growth and cancer aggressiveness [161,218].

## 7. Conclusions

This review summarizes the most recent research advances on the role of neuroglial LDs in the function of the CNS and in neurologic disorders. LDs tend to accumulate in neuroglia in the brain as a response to neuronal hyperactivity during development and aging, as well as in neurologic disorders. Alterations in LD metabolism in neuroglia have been linked to metabolic and oxidative stress, inflammation, disease-related gene mutations, and stress-related adrenergic activation. The accumulation of lipids in neuroglial LDs has been shown not only to protect stressed cells against lipotoxicity, free radicals (antioxidant role), and nutrient deprivation, thus enhancing cell viability, but also to potentially contribute to disease development, as has been suggested for gliomas and neurodegeneration. Although there are still many gaps in our understanding of the molecular mechanisms underlying the alterations in neuroglial LD metabolism and how they affect neural function in health and disease, these novel findings highlight neuroglial LDs as a potential therapeutic target in aging and neurologic disorders.
